# Sex and Gender Differences in Chronic Kidney Disease—Explained by the Brenner–Luyckx Concept of Hyperfiltration

**DOI:** 10.3390/jcm15041654

**Published:** 2026-02-22

**Authors:** Sylvia Stracke, Jonas Wille, Angelina Smolka, Ron Henkel, Kirubel Biruk Shiferaw, Dagmar Waltemath, Frieder Keller, Tilman Schmidt, Robert Wolf, Thomas Dabers, Till Ittermann, Philipp Töpfer

**Affiliations:** 1 Nephrology, Internal Medicine A, University Medicine Greifswald, 17475 Greifswald, Germany; jonas.wille@med.uni-greifswald.de (J.W.); angelina.smolka@med.uni-greifswald.de (A.S.); tilman.schmidt@med.uni-greifswald.de (T.S.); robert.wolf@med.uni-greifswald.de (R.W.); thomas.dabers@med.uni-greifswald.de (T.D.); philipp.toepfer@med.uni-greifswald.de (P.T.); 2 KfH Renal Center, 17475 Greifswald, Germany; frieder.keller@uni-ulm.de; 3 Medical Informatics Laboratory, University Medicine Greifswald, 17475 Greifswald, Germany; ron.henkel@uni-greifswald.de (R.H.); kirubelbiruk.shiferaw@med.uni-greifswald.de (K.B.S.); dagmar.waltemath@uni-greifswald.de (D.W.); 4 Institute for Experimental and Clinical Pharmacology, University Hospital Ulm, 89081 Ulm, Germany; 5 Institute for Community Medicine–SHIP Clinical-Epidemiological Research, University Medicine Greifswald, 17475 Greifswald, Germany; till.ittermann@uni-greifswald.de

**Keywords:** chronic kidney disease, hyperfiltration theory, sex differences, gender differences, social determinants of health, clinical practice guidelines

## Abstract

At the beginning of life, there are no sex differences in fetal kidney growth, nephron endowment nor in the prevalence of low birth weight. In chronic kidney disease (CKD) in adults, however, significant sex- and gender-specific differences exist in diagnosis, progression, and management of CKD. In adult individuals, CKD is more prevalent in women, but CKD progression is faster in men; nevertheless, women have a higher life expectancy than men. A possible explanation for the enigmatic higher CKD prevalence in women may derive from the Brenner–Luyckx concept of hyperfiltration. Diseases that lead to hyperfiltration will lead to premature nephron loss and to a faster decline in glomerular filtration rate (GFR). This condition is predominantly seen in middle-aged men with a higher GFR, larger hypertrophied kidneys, and a higher prevalence of arterial hypertension, diabetes mellitus, smoking, and hypercholesterolemia compared to women. Thus, a high GFR may not be a good sign if it reflects hyperfiltration. Any GFR must be interpreted against the comorbidities of an individual. An individual may end up with a realistic GFR far below normal once hyperfiltration is stopped, for example, by a Sodium Glucose-Linked Transporter 2 (SGLT2) inhibitor. With regard to the management of CKD, women with CKD receive poorer healthcare compared to men with CKD. Women less frequently receive a CKD diagnosis, are less frequently referred to nephrology for co-management, less frequently undergo eGFR and albuminuria assessments, and are less likely to receive guideline-recommended treatments for CKD, such as angiotensin-converting enzyme (ACE) inhibitors, angiotensin receptor blockers, and statins. Cardiovascular risk factors are less rigorously controlled in women with CKD compared to men with CKD. The causes for the poorer CKD care among women are to be found in gender rather than in sex. It is crucial to integrate assessments of sex and gender into both clinical routines and scientific reports. All studies should incorporate sex- and gender-specific analyses, and the evaluation of pre- and postmenopausal women should be conducted separately. The utilization of Gender Scores can help identify the impact of cultural, societal, and psychological factors on observed gender differences in ambulatory healthcare for those with CKD. Guidelines need to be sensitive to gender and emphasize the existing knowledge gaps regarding sex and gender differences in CKD healthcare. Urgent attention is required to substantially improve and ensure equitable healthcare for CKD across sexes and genders.

## 1. Sex and Gender in Medicine: Implications for the Care of Chronic Kidney Disease Patients

Epidemiological indicators, pathophysiology, symptoms, natural courses, therapy response rates, and side effect profiles of medications show significant sex and gender differences in many common diseases, including chronic kidney disease (CKD) [1,2]. Sex refers to biological sex, considering anatomical, genetic, and hormonal differences assigned at birth. The social and psychological construct of gender encompasses attitudes, feelings, and behaviors associated with gender assigned at birth, both by the individual and by society. Neglecting or inadequately considering sex and gender aspects in medical research and practice can have serious consequences for diagnosis, treatment, or risk prediction [2,3]. For instance, women compared to men may experience a higher rate of medication side effects [4], poorer response to immunotherapies in oncological conditions [5], higher cardiovascular risk despite guideline-compliant treatment of hypertension [6], and suboptimal care for CKD [3]. Throughout their lifespan, women are exposed to various factors influencing cardiovascular and renal health, which have not been systematically investigated in this context and have not been adequately addressed in guidelines for the management of CKD, diabetes, and hypertension.

Throughout this review, we follow three interwoven concepts:

(1) the absence of sex differences in kidney development and nephron number;
(2) sex differences in adult eGFR and kidney size driven by hyperfiltration;
(3) differences in CKD prevalence and progression primarily attributable to gender-related (social and behavioral) factors.

## 2. The Beginning of Life: No Sex Differences in Kidney Development and Nephron Numbers

### 2.1. No Sex Difference in Fetal Kidney Growth

During regular human kidney development, nephron formation concludes by term birth, with the majority of nephrons formed late in gestation. A study of human fetal kidneys revealed that kidney growth in the second half of gestation is directly proportional to body weight and gestational age in both male and female infants [7]. Female infants were significantly lighter in weight overall, with significantly reduced kidney weight in comparison to male infants. However, the kidney weight to body weight ratio remained constant throughout the gestational period with no sex differences [7]. These findings align with previous research indicating that, towards the end of gestation, male infants tend to have a higher birth weight than females at the same gestational age, and kidney size during development (length and volume) is smaller in females compared to males [8,9]. Importantly, the study highlights that the growth of kidneys (weight gain over time) in the second half of gestation is not different between the sexes [7], but there was variation in the timing of the cessation of nephrogenesis and in the number of glomerular generations formed [7].

### 2.2. No Sex Difference in Low Birth Weight and Associated Reduced Nephron Numbers: Developmental Programming

Low birth weight (LBW), as defined by the World Health Organization (birth weight < 2500 g), and adult cardiovascular disease have long been linked to poor fetal growth and a compromised feto-maternal environment [10,11,12]. Studies from diverse populations corroborate these findings, encompassing LBW-related conditions, such as hypertension, type 2 diabetes, obesity, and CKD [13]. The term developmental programming describes the longitudinal structure–function effects experienced during critical periods of fetal and early postnatal growth in response to environmental stimuli [12,14]. Developmental programming may represent the initial phase in a sequence of intra-uterine events that eventually manifest as overt disease. LBW can result from intra-uterine growth restriction or preterm birth, with term LBW exhibiting the strongest association with adult disease [15]. In nephrology, LBW, small for gestational age, and prematurity have been demonstrated to associate with an increased risk of albuminuria [16], hypertension [17], chronic kidney disease [18], and kidney failure [19].

LBW is also associated with an increased risk of developing focal segmental glomerulosclerosis (FSGS) in later life [20]. Adult patients with FSGS show a higher prevalence of LBW compared with the general population supporting the concept that reduced nephron endowment at birth confers long-term susceptibility to post-adaptive glomerular injury. This association is particularly evident in secondary FSGS, where glomerular hypertrophy and hyperfiltration are predominant pathological features [20,21]. The association between LBW and FSGS is mechanistically explained by the developmental programming hypothesis of kidney disease. Luyckx emphasizes the link between impaired fetal growth and early life conditions to long-term kidney risk on a global scale leading to an appeal to improve maternal nutrition, antenatal care, and early childhood health worldwide [22].

**Figure 1:**
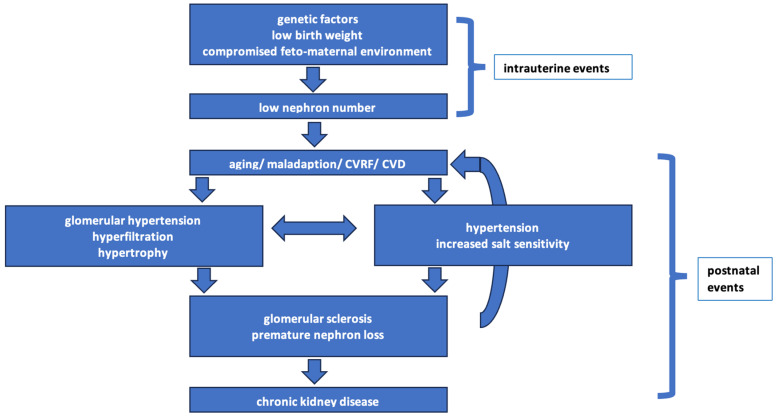
The Brenner–Luyckx concept of hyperfiltration: a low nephron number at birth leads to glomerular hyperfiltration and overload of the remaining nephrons starting a vicious cycle. Even with normal nephron numbers, factors leading to hyperfiltration, such as (pre-)hypertension, (pre-)diabetes, smoking, and hypercholesterolemia, will enter the cycle at a later stage but still contribute to the same mechanism ending in premature nephron loss. Men have a higher prevalence of these conditions, and their higher eGFR may rather reflect a hyperfiltrational state than a better renal function compared to women. Nephron overload and hyperfiltration are newly recognized therapeutic targets for nephron protection and slowing CKD progression (adapted from [24,38,39,40]).

According to the Brenner–Luyckx concept of hyperfiltration, impaired intra-uterine growth results in a reduced nephron number, which leads to life-long compensatory hyperfiltration, increased intraglomerular pressure, podocyte depletion, and, ultimately, to glomerulosclerosis (Figure 1). This framework provides a unifying explanation linking LBW to hypertension, CKD, and secondary FSGS in later life [14,23,24].

Thus, it is believed that a low nephron number at birth is compensated by hyperfiltration of the remaining nephrons [24,25,26]. Substantial evidence supports the connection between LBW and renal dysfunction in humans demanding attention directed towards maternal health before and during pregnancy. In a cross-sectional analysis of the National Health and Nutrition Examination Survey, there was no sex difference seen in the prevalence of LBW (<2500 g) [27].

### 2.3. No Sex Differences in Nephron Endowment

Renal cortical volume decreases with age, but medullary volume increases with age [28]. Therefore, cortical volume serves as a potential surrogate for detecting the decline in nephron numbers. In a study involving 1638 living kidney donors, the total kidney parenchymal volume was segmented, and cortical volume was subsequently estimated from the total kidney parenchymal volume in CT scans [29]. At the time of kidney transplant, sections of a biopsy specimen of the cortex were obtained, and the total number of glomeruli was estimated from cortical volume and glomerular density. Donors aged 18–29 years exhibited a mean of 990,661 nonsclerotic glomeruli and 16,614 globally sclerotic glomeruli per kidney, which progressively decreased to 520,410 nonsclerotic glomeruli per kidney and increased to 141,714 globally sclerotic glomeruli per kidney in donors aged 70–75 years. Nephron loss with aging, whether through increased glomerulosclerosis or cortical volume decline, is consistent with the atrophy and reabsorption of globally sclerotic glomeruli and the hypertrophy of remaining nephrons. A lower nephron number in healthy adults, associated with characteristics indicative of both lower nephron endowment at birth and subsequent nephron loss, is reflected in clinical features. Factors independently associated with a lower nephron number include older age, shorter height, a family history of kidney failure, higher uric acid level, and lower GFR. Interestingly, sex was not associated with a lower nephron number after multivariable adjustment (Supplemental Table S2 in [29]).

## 3. The Brenner–Luyckx Concept of Hyperfiltration: Implications for Sex- and Gender-Specific Outcomes in CKD

### 3.1. The Brenner–Luyckx Concept: Nephron Number, Arterial Hypertension and Hyperfiltration

In 1986, Barker et al. were pioneers in demonstrating that adults with a history of low birth weight faced a higher risk of cardiovascular disease [30]. Building on this, in 1988, Brenner proposed a hypothesis suggesting an inverse relationship between nephron number and hypertension [26]. Brenner and colleagues postulated that developmental programming in the kidney might diminish nephron number, subsequently contributing to arterial hypertension in later life [26]. Throughout a person’s lifetime, it is anticipated that even healthy individuals will experience a substantial loss of nephrons as part of the normal aging process. Therefore, a low nephron endowment at birth, combined with age- or disease-related nephron loss, could predispose individuals to the development of hypertension [31]. Early support for the Brenner hypothesis came from studies indicating significantly lower nephron numbers in individuals previously diagnosed with hypertension compared to those in normotensive individuals [32,33,34]. However, it is difficult to measure total nephron number in utero, at birth, and throughout an individual’s lifespan in humans as there are few imaging modalities available to adequately assess fetal nephron endowment and postnatal nephron numbers in vivo [35,36]. Most reported measures of nephron number in humans also involve indirect measures of nephron number, such as kidney mass or volume [37]. Kidney biopsy is inadequate for extrapolating total nephron numbers, as methods employing the whole kidney provide the most accurate measurement of nephron number [31].

In summary, Brenner proposed the hypothesis that low birth weight infants are born with impaired renal development, resulting in a reduced nephron number. This reduction, in turn, leads to systemic and glomerular hypertension, forming a vicious cycle that gradually progresses to the eventual development of hypertension in adulthood. The hypothesis also suggests that a reduced nephron number and total glomerular filtration surface area are associated with the development of hypertension.

### 3.2. Hyperfiltration—Sex- and Age-Specific Reference Values for Kidney Size and eGFR

This section was thought to address sex-related biological mechanisms—but finally led to the idea of gender-related mechanisms.

Research has indicated that age-related structural changes in the kidneys differ between men and women [28,41,42]. In women, kidney size appears to remain stable until around the fourth decade before a decline begins, while in men there seems to be an increase until approximately the fifth decade before a decline sets in [43,44]. A recent population-based MRI study conducted on 1815 individuals from Northeast Germany revealed an inverse U-shaped association of kidney size with age in males [42]. Cardiovascular risk factors, such as diabetes, hypertension, and smoking, were more prevalent in men, and it was assumed that these factors mediate hyperfiltration, renal hypertrophy, and increased kidney size in middle-aged men [42]. For the same eGFR, women’s kidneys were smaller than men’s kidneys [42]. The larger renal volumes at the same eGFR in men raises again the question of hyperfiltration and hypertrophy with subsequent faster CKD progression in men in the long term. The clinical relevance of glomerular hyperfiltration was also shown in a recent retrospective cohort study, which enrolled 1,952,053 patients with type 2 diabetes mellitus from the Korean National Health Insurance Service database between 2015 and 2016. Hyperfiltration was associated with cardiovascular disease (CVD), particularly myocardial infarction and heart failure, and the impact of glomerular hyperfiltration was greater in younger patients [45].

The physiological loss of kidney function was examined in another population-based longitudinal study involving 1837 middle-aged individuals (50–62 years) with healthy kidneys, using Iohexol clearance measurements [46]. At the beginning, women had a lower measured glomerular filtration rate (mGFR) compared to men (90.0 + 14.0 mL/min vs. 98.0 + 13.7 mL/min; *p* < 0.001). However, over the observation period, men experienced a 25% steeper decline in mGFR compared to women (1.20 mL/min per year in men vs. 0.96 mL/min per year in women). This aligns with the assumption of hyperfiltration due to a higher prevalence of cardiovascular risk factors and cardiovascular disease in men compared to women.

### 3.3. Definition of Hyperfiltration and Differential Interpretation

Also, this paragraph was meant to address binary (male/female) sex-related biological mechanisms and again led to the idea of gender-related and, therefore, modifiable mechanisms.

**Figure 2:**
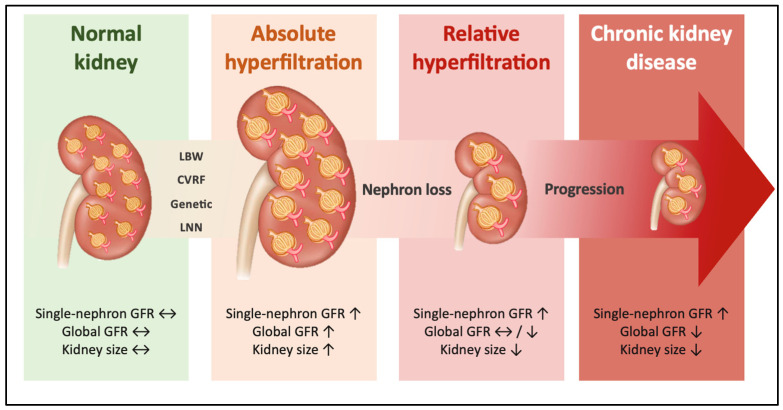
Different states of hyperfiltration and their effects on single-nephron GFR, global GFR, and kidney size (↑ = increase, ↓ = decrease, ↔ = no change). LBW: low birth weight; CVRFs: cardiovascular risk factors; LNN: low nephron number (adapted and modified from [49]).

Absolute hyperfiltration is defined by an eGFR above the sex- and age-specific 95th percentile for healthy individuals [47]. Absolute hyperfiltration initially induces compensatory glomerular hypertrophy; however, exhaustion of these adaptive mechanisms ultimately results in progressive nephron loss (Figure 2) [48]. Hyperfiltration at the level of individual nephrons, which leads to increased single-nephron GFR, cannot automatically be translated into global GFR due to existing nephron loss; consequently, global GFR may be either normal or reduced, a condition known as relative hyperfiltration (Figure 2) [48].

The study conducted by Okada et al. in 2012 [47] included 99,140 individuals aged 20–89 years who underwent health checkups in Aichi Prefecture, Japan. The prevalence of hyperfiltration (eGFR above the age-/sex-specific 95th percentile) and hypofiltration (eGFR below the age-/sex-specific 5th percentile) was compared among stages of prediabetes (fasting plasma glucose < 100, 100–109, 110–125, and 126 mg/dL for no prediabetes, stage 1 prediabetes, stage 2 prediabetes, and diabetes, respectively) and prehypertension [blood pressure (BP) < 120/80, 120–129/80–84, 130–139/85–89, and 140/90 mmHg for no prehypertension, stage 1 prehypertension, stage 2 prehypertension, and hypertension, respectively]. The prevalence of hyperfiltration increased with the advancing stage of prediabetes (Odds Ratios [ORs]: 1.29, 1.58, and 2.47 for stage 1 prediabetes, stage 2 prediabetes, and diabetes, respectively) and prehypertension (ORs: 1.10, 1.33, and 1.52 for stage 1 prehypertension, stage 2 prehypertension, and hypertension, respectively). Hypofiltration was not associated with prediabetes or prehypertension. Therefore, any eGFR must be interpreted against the comorbidities of the individual. A “normal” eGFR might not truly be normal, as any eGFR can coexist with a hyperfiltrational state. The nomogram presented by Okada in 2012 [47] indicates that (1) there is no sex difference in eGFR in healthy subjects, and (2) from the age of 60 years, an eGFR <60 mL/min is within the “normal filtration range” for both men and women.

So far, hyperfiltration has not entered the classification stages of KDIGO CKD definition. According to KDIGO, anyone with an eGFR below 60 mL/min is defined to suffer from chronic kidney disease. In the subsequent paragraphs, we will employ the KDIGO definition to report on CKD prevalence and progression and see that this will lead to women having higher CKD prevalence and men showing faster CKD progression despite having larger kidneys and a seemingly “better” eGFR. LGBTQI+ individuals are underrepresented in most trials, but so far, CKD and CVD seem to be less prevalent in these cohorts, considering that they usually consist of younger individuals.

We here suggest a new concept of hyperfiltration causing CKD and explaining sex and gender differences in eGFR—however, much of the supporting evidence is derived from observational studies. Whether hyperfiltration represents a causal mechanism or a consequence of underlying disease remains debated and causality has not been definitively established. Still, hyperfiltration currently represents the most coherent explanatory hypothesis for the observed phenomena.

## 4. Worldwide Prevalence of Chronic Kidney Disease in Women, Men, and LGBTQI+ Individuals

### 4.1. CKD Prevalence in Women and Men

This section addresses binary (male/female) sex-related biological mechanisms.

CKD represents a significant public health issue. CKD is classified in five stages according to eGFR and albuminuria (ACR). The terms hypo- or hyperfiltration are not part of this classification system. All GFR estimation formulas (BIS, CKD-EPI, EKFC, FAS, MDRD, etc.) include age and sex. It is not easy to explain why—after correcting for sex in each of those formulas—women still have a higher CKD prevalence compared to men: The prevalence of CKD in the general population in Europe and the USA varies between 10 and 20% [50,51,52,53]. While the prevalence of CKD in adult U.S. men from 2017 to 2020 was 12.6%, women showed a significantly higher CKD prevalence of 15.4% during the same period [52]. A similar difference was observed in the German Health Study of Adults (DEGS1) among individuals over 70 years old. Here, CKD prevalence in women was 15%, compared to 11% in men [51]. In other countries, such as France (12.6 vs. 6.4%), Portugal (7.8 vs. 3.7%), and Thailand (12.3 vs. 5.5%), a higher CKD prevalence in women compared to men was also noted [1]. Only in Japan, CKD prevalence is higher in men (15.7%) than in women (11.7%) [1].

In the European Chronic Kidney Disease Burden Consortium which comprises nine general population-based studies from seven European countries providing European age- and sex-specific eGFR reference values in healthy adults using the European Kidney Function Consortium (EKFC) equation, 2,572,020 individuals were included of which 1,535,253 (60%) were considered healthy (45% male, 55% female) [54]. The study showed that eGFR was not preserved with aging in healthy individuals. Creatinine-based eGFR in this study was lower at higher ages with a slightly steeper eGFR–age decrease in women than men, leading to a lower eGFR in women compared with men after the age of 60 years [54].

The difference in CKD prevalence between women and men is not well explained at all, especially considering that women have a higher life expectancy than men. A poorer eGFR is associated with higher mortality in most studies. One explanation for the higher CKD prevalence in women is a longer life expectancy with only moderately impaired renal function in older age, reflecting physiological loss of renal function with age. The physiological loss may have a different clinical relevance for women compared to men. It is also possible that the GFR estimation formulas used so far may overdiagnose CKD in women. Both the CKD-EPI and MDRD formulas were not developed in the general population. The CKD-EPI formula underestimates the measured GFR in both genders [55], but more frequently in women than in men (mean difference mGFR-eGFRcrea 14.2 mL/min in women vs. 3.4 mL/min in men). Therefore, defining sex-specific thresholds for CKD classification seems reasonable [56].

A new explanation for the enigmatic higher CKD prevalence in women is that the putatively good (higher) eGFR in men is not good, but indicative of a hyperfiltrational state. Possibly, new thresholds for men and women in a healthy renal state could be considered—excluding individuals with any CVRFs, such as smoking, hypercholesterolemia, arterial hypertension and diabetes, and any CV disease to exclude measuring potential hyperfiltration.

In diabetes, both hyperfiltration and the inhibition of hyperfiltration by SGLT2-inhibitors are well known [39]. The reduction in hyperfiltration ultimately slows the progression of DKD and CKD of any origin.

Finally, age- and sex-adapted thresholds for defining CKD have been recently proposed based on the lower 5th and 2.5th percentile of eGFR reference values derived from over 1.5 million healthy individuals from a multinational European cohort [57]. Compared with the KDIGO eGFR threshold, the study showed a higher prevalence of reduced eGFR in younger adults and a lower prevalence in older adults when applying age-adapted eGFR thresholds. In older adults, the CKD prevalence was lower for females compared to males when using the age-adapted eGFR thresholds [57].

### 4.2. CKD Prevalence in LGBTQI+ Persons

This section focuses on gender-related social and behavioral factors.

Currently, approximately 0.5% of people worldwide identify as transgender [58]. However, as only about half of transgender individuals disclose this fact to their healthcare providers, the actual prevalence is likely to be higher. Around 80% of transgender individuals use gender-affirming hormone therapy [58]. There is limited literature on the effects of this therapy or of gonadectomy on kidney function and CKD prevalence.

In a U.S. cross-sectional study involving 346,868 individuals from the “All of Us Research” study, 8.9% identified as LGBTQI+ [59]. This group, compared to cisgender heterosexual individuals, had higher Odds Ratios (OR) for anxiety disorders, depression, and nicotine abuse but mostly lower OR for CKD, cardiovascular diseases, diabetes, and hypertension [59]. Also, in this setting, the prevalence of CKD is associated with CVRFs and CVD; therefore, the Brenner–Luyckx’s concept of hyperfiltration may also explain the findings seen in the LGBTQI+ population from the “All Of Us” cohort. Exceptions regarding CKD prevalence within the LGBTQI+ cohort include cisgender homosexual men (CKD with an OR of 1.06 vs. cisgender heterosexual men) and transgender women (CKD with an OR of 1.11 vs. cisgender heterosexual women) (Table 1).

The available data on LGBTQI+ persons are limited by younger age distributions, predominantly cross-sectional study designs, and low numbers of CKD events. The reported findings are hypothesis-generating, and no definite conclusions can be drawn until longitudinal data become available.

## 5. Sex- and Gender-Specific Differences in CKD Progression and Potential Protective Mechanisms

### 5.1. Sex- and Gender-Specific Differences in the Prevalence of Risk Factors and the Progression of CKD

This section starts with addressing sex-related differences and closes with gender-related social and behavioral factors.

Many population-based studies describe a faster progression of CKD in men compared to women. However, whether CKD progression is indeed lower in women than in men is not definitively supported by current studies [1]. Two older large meta-analyses presented divergent results. In Neugarten et al.’s meta-analysis, CKD progression was examined in 68 cohort studies of the general population with non-diabetic CKD [60]. Here, CKD progression was more pronounced in men than in women. In another meta-analysis based on 11 Randomized Controlled Trials (RCTs) assessing the use of ACE inhibitors in CKD, this sex-specific difference in CKD progression was not observed [61]. The absence of sex-specific differences in the latter meta-analysis was attributed to the majority of women being postmenopausal. However, most studies lack data on the hormonal status of women, except for the age of participants as a surrogate for postmenopausal status.

The prevalence of classical cardiovascular risk factors—such as diabetes, hypertension, smoking, and hypercholesterolemia—is different between the sexes, with higher prevalence in males leading to hyperfiltration and larger kidneys in middle-aged and a faster eGFR decline in old-aged men [42,56]. In a recent study on 1916 participants (983 females) aged 21 to 81 years from the Study of Health in Pomerania, we observed associations of arterial hypertension with larger renal volumes (measured by magnetic resonance imaging) up to the age of 55 and with smaller renal volumes at older ages. These associations were seen in both sexes but were stronger in males than in females [62]. The accumulation of cardiorenal risk factors, first leading to renal hyperfiltration and hypertrophy and later to an accelerated parenchymal loss, seemed to be a plausible explanation for this phenomenon.

Another CKD risk factor is thyroid function: hypothyroidism is associated with reduced kidney function [63]. In multivariable analyses of cross-sectional data from 7933 individuals aged 20 to 93 years from the Berlin Aging Study II and the Study of Health in Pomerania, an inverse non-linear association of serum TSH levels with eGFR was described, while serum fT3 levels showed a positive association with eGFR. Both high and low serum fT4 levels were associated with a lower eGFR. While age modified the association between thyroid hormone levels and eGFR, sex did not. Nonetheless, hypothyroidism is more prevalent in women compared to men [63].

Additionally, there are new CKD risk factors, such as the use of proton pump inhibitors (PPI) versus histamine receptor antagonists (H2RA) [64]. Chen et al. describe that dose-dependent H2RA use was associated with a reduced risk of kidney failure and overall mortality in CKD patients, whereas PPI use was associated with an increased risk of overall mortality, although not in a dose-dependent manner [64]. These effects of H2RA were present in both males and females (Supplemental Table S3 in [64]). However, polypharmacy and the use of PPI are more prevalent in women compared to men [65].

Renal function decreases with age and nephrologists are confronted with a large cohort of old and very old patients, as described by Torreggiani et al. 2021 [66]. In their cohort of 1992 patients referred to a university renal center, patients > 60 years accounted for about 70% of the overall cohort, about 25% were aged ≥80 years, and 6% were ≥90 years. This referral pattern of CKD patients to nephrology means that female CKD patients are mostly postmenopausal and that this aspect should be taken into account. The referral pattern was characterized by referrals of older patients with lower eGFR. The use of different formulas did not lead to a relevant difference between patients with eGFR above or below 45 mL/min, the threshold now suggested for the definition of CKD in the elderly [66]. This study did not provide sex-specific analyses. However, as females have a longer life expectancy, more women of old age will experience KDIGO CKD stages 3–5, and they will especially benefit from new age- and sex-specific eGFR thresholds. They will no longer be defined as suffering from CKD but, instead, show adequate renal function according to age- and sex-specific thresholds.

Additional parameters of interest that are not routinely collected include pre/postmenopausal status, number of pregnancies with/without pregnancy-induced hypertension/diabetes/preeclampsia, use of hormonal contraceptives, hormone replacement therapy, use of estrogen receptor modulators in breast cancer, the use of gender-affirming hormone therapy in transgender individuals, and the individuals’ own views on their gender [58,67,68]. Furthermore, gender-specific aspects of CKD may be influenced by both biological (sex) and socially influenced gender differences. The lower OR for CKD, cardiovascular diseases, diabetes, and hypertension in LGBTQI+ individuals, as well as the longer life expectancy of women despite lower eGFR, point to gender rather than sex differences [59]. Hyperfiltration, mostly seen with a higher burden of modifiable cardiovascular risk factors (such as smoking, higher body weight associated with (pre)diabetes and (pre)hypertension, metabolic syndrome, smoking), is a matter of gender—not sex.

Therefore, it seems reasonable to (1) encode sex and gender information in clinical and epidemiological studies from the beginning according to the “Sex And Gender Equity in Research” (SAGER) guidelines [69] and (2) establish Gender Scores and apply these to all studies instead of only reporting sex differences as also planned in a study called “Inclusive Excellence in Medicine” funded by the German Federal Ministry of Research, Technology and Space [67,68].

### 5.2. Gender Scores and the Influence of Gender on CKD and Cardiovascular Diseases

This section focuses on gender-related social and behavioral factors.

To capture the societal, cultural, and psychological influences of gender on the care of individuals with CKD, Gender Scores are used. Ideally, gender is assessed at study enrollment using tools such as the Bem Sex Role Inventory (BSRI) questionnaire [70]. However, in most studies, only sex has been recorded in binary form. Alternatively, Gender Scores can be developed from existing data. In two large Canadian population studies, variables related to childcare, employment, weekly working hours, household income, personal income, education level, and relationship status were used to create a Gender Score [71,72].

Country-specific Gender Scores were developed in two Canadian–European studies [73,74]. A higher score in these studies indicated that the individual exhibited more traits traditionally associated with the female gender. The GOING-FWD Consortium (Gender Outcomes INternational Group: to Further Wellbeing Development), which investigates the influence of gender on chronic diseases, involves participants from Canada, Italy, Spain, Sweden, and Austria. Regardless of sex, individuals with diabetes mellitus and a higher (more “feminine”) Gender Score had poorer diabetes management and worse cardiovascular outcomes [74]. In countries with a higher Gender Inequality Index, reflecting stronger institutionalized gender inequality, this effect was more pronounced. The United Nations’ Gender Inequality Index includes variables such as maternal mortality, birth rate among 15–19-year-olds, the proportion of women in parliament seats, the proportion of women and men with secondary education, and employment rates. The political dimension of the question of gender-equitable care for individuals with CKD becomes evident here.

Similar to Gender Scores, there are also Masculinity and Femininity Scores, each showing clinically relevant effects in the interpretation of studies. These scores also include variables from the domains of occupation/education, caregiving, lifestyle, and emotions. Male gender, as indicated by a higher Masculinity Score, was associated with a lower prevalence of chronic diseases—both in males and females with a high Masculinity Score [65,75]. For chronic diseases with a higher prevalence in men (diabetes, coronary heart disease, peripheral arterial disease), the gender difference increased when adjusted for gender. For example, the OR for diabetes changed from 1.21 to 1.60 when adjusted for gender. This means that the effect of sex is underestimated, and not accounting for gender in studies understates the effect of sex. For chronic diseases with a higher prevalence in women (arthritis, chronic pain syndrome, migraine), the gender difference became smaller or disappeared altogether when adjusted for gender. In these diseases, there would be no sex difference if there were no gender, as exemplified by arthritis, where the OR increased from 0.53 to nearly 1.0. Gender can thus be seen as a mediator of sex differences in chronic diseases [75].

Similar influences are reported in the GENESIS-PRAXY study when applying a Gender Score [76]. In multivariable models that included both sex and a Gender Score in the analyses, a “feminine” Gender Score, but not female sex, was associated with the presence of cardiovascular risk factors.

### 5.3. Sex- and Gender-Sensitive Study Participation and Lack of Knowledge Not Reported in Clinical Guidelines

Randomized Controlled Trials (RCTs) are the gold standard in clinical research, yet there are distinct gender-specific differences within them. Many studies focus on average effects in a selected population and rarely report results of sex- or gender-specific analyses, despite capturing binary sex data. Without sex- and gender-specific analyses, the external validity and generalizability of studies are limited. Reliable sex- and gender-specific reporting requires a balanced participation of women, men, and LGBTQI+ individuals in clinical trials. Despite constituting 55% of the global CKD population, a recent review of 192 CKD studies with a total of 150,000 participants revealed that only 45% of study participants were women [77]. In current studies on the use of SGLT2 inhibitors in CKD, women’s participation is even lower at 33% [78]. The underrepresentation of women in clinical trials is particularly evident in Europe [77]. The issue appears to affect recruitment, as dropout rates in studies are hardly different between men and women [79]. A recently published report on over 600 international studies in cardiovascular diseases suggests that studies led by women are more likely to recruit women into the studies [80]. The underrepresentation of women as study leaders and participants potentially poses an obstacle to drug assessment. In the meta-analysis by Pinho-Gomes et al. [77], sex-specific results for drug efficacy assessments were presented in only one-fifth of the studies, and none of the 192 included studies considered sex-specific differences in safety assessments. When including women in studies, it should also be considered whether they are pre- or postmenopausal, taking hormones, and whether they have experienced pregnancy-related conditions influencing kidney function [56].

Thus, despite global guidelines and editorial policies, integration of sex and gender analyses remains limited in health research [81]. Women in senior authorship positions are more likely to produce research articles that include and discuss sex and gender differences [82]. Although RCTs in the field of dialysis are representative of the general dialysis population with regard to sex, sex is often not included neither in reporting nor in analyses [83]. In recent landmark kidney trials examining SGLT-2 inhibitors, GLP1-receptor antagonists, and the nonsteroidal MRA, finerenone, women remain significantly underrepresented compared with male trial participants [84].

There is scarce population-based data on LGBTQI+ individuals, mainly because data on gender diversity, sexual orientation, and gender identity are not routinely collected. It makes sense to plan studies as gender-diverse studies and use validated instruments to capture all gender identities and sexual orientations [58,67,68,85]. This is, however, a difficult task. So far, sex and gender are not included in CONSORT (Consolidated standards of reporting trials/Cochrane) neither in 2010 nor in the 2022 extension [86]—although this had already been criticized in 2012 [87]. Also, NIH policies have not resulted in significant increases in reporting results by sex or ethnicity [88]. Instead, it has become even worse: in 2015, 77% of studies did not include sex in analysis nor provided an explanation for this behavior [88].

It has to be stated that data sources are not real—sex and gender are neither recorded nor reported: there is an urgent need to standardize the way sex and gender are reported—as already required by many national funding agencies for proposed projects (e.g., US, Canada, Europe).

The recommendations suggested by the Sex and Gender Equity in Research (SAGER) guidelines comprise [69]:

- use the terms sex when reporting biological factors and gender when reporting gender identity or psychosocial or cultural factors;
- disaggregate demographic and all outcome data by sex, gender, or both;
- report the methods used to obtain information on sex, gender, or both;
- note all the limitations of these methods.

If we do not comply with the SAGER standards, we end up with confusing results in clinical practice and with clinical practice guidelines that are potentially harmful for those underrepresented or underreported in clinical trials: women (pre-, peri- and postmenopausal women) and LGBTQI+ persons.

So far, there are no sex- or gender-specific guidelines in nephrology, although according to KDIGO CKD classification, females are 30% more likely to have CKD. A systematic review on “Integration of Sex and Gender in Trials for Chronic Kidney Disease (INSiGhT_CKD)” is ongoing [89]. European CPGs in internal medicine specialties including nephrology focus only marginally on sex/gender-related aspects, guideline committees lack gender balance and context sensitivity, and the applicability of the CPGs beyond high income country settings is not given [90].

Women have different CKD and CV risk factors and complications [91]. The prevalence of hypertension is lower in premenopausal women than in men of the same age, but sharply increases after menopause, resulting in higher rates in women aged 65 and older. Female-specific factors in (patho)physiology and treatment response comprise beneficial CV effects of estrogen, such as vasorelaxation, inhibition of the sympathetic nerve system, and prevention of vascular remodeling [92]. Estrogen also alters the balance between the vasoconstricting and vasodilating effects of the renin–angiotensin–aldosterone system (RAAS), favoring vasodilation. After menopause, these beneficial effects disappear and the arterial wall rigidity increases. Due to a shorter stature, women have a shorter arterial tree than men, which may lead to the amplification of peak systolic blood pressure by the reflected waves [93]. Female-specific causes of hypertension comprise polycystic ovary syndrome, use of contraceptive agents, assisted reproductive techniques, pregnancy-related hypertensive disorders, and premature ovarian insufficiency, e.g., due to breast cancer treatment [93,94]. Additionally, in women, CV risk increases at a lower blood pressure compared to men (already at 130/80 mmHg) [6].

## 6. Conclusions

The hyperfiltration theory is derived from the Brenner–Luyckx concept that a low nephron number at birth leads to hyperfiltration of the remaining nephrons and to arterial hypertension in later life. Glomerular hyperfiltration and hypertrophy mean temporary larger kidney volumes and temporary higher GFR—which may not be a good sign but indicative of future faster CKD progression. The hyperfiltration theory challenges the KDIGO CKD classification. KDIGO defines CKD as GFR < 60 mL/min. We may need to change our mindset, especially in the light of the use of SGLT2-inhibitors where it is accepted to continue treatment even when the eGFR is dropping towards the putative real GFR—once the kidneys are freed from the burden of hyperfiltration. Hyperfiltration mostly seen with a higher burden of modifiable cardiovascular risk factors is a matter of gender—not sex. Still, we are aware that these are perspective-driven observations that include many unresolved issues and the need for future validation.

We then need new studies including all genders in a healthy renal state—excluding individuals with any CVRFs, such as smoking, hypercholesterolemia, arterial hypertension, and diabetes, and any CV disease to exclude measuring potential hyperfiltration. We will only then see whether we also need sex-specific thresholds for GFR. At the moment, the higher prevalence of CVRFs and CVD in men compared to women seems to confound the interpretation of GFR measurements. Any GFR must be interpreted against the comorbidities of the individual, and nephron overload must be addressed as a therapeutic target for all genders. Studies during kidney development show no sex difference in growth of the kidneys nor in nephron endowment. Lower nephron number and low birth weight on the other hand are associated with CKD and hypertension in later life. To reduce the number of babies born with low birth weight, maternal health before and during pregnancy should be addressed [24].

## 7. Future Directions

None of the female-specific causes for CKD or hypertension, nor LGBTQI+ individuals’ health, nor the fact that the hyperfiltration theory may challenge the KDIGO CKD classification have been taken into account in current clinical practice guidelines in nephrology. This lack of knowledge should, at least, be mentioned, and further research directions need to be discussed.

Data quality has to be improved as in many studies sex and gender information are either not collected or not analyzed or both. Sex and gender equity in kidney research and clinical practice is mandatory to address all individuals’ kidney health issues and to finally achieve individual and personalized nephrological care. The following is claimed:

1. Encode sex and gender information in clinical and epidemiological studies from the beginning according to SAGER standards.
2. We suggest that gender (identity, but also roles and relations) is encoded at the beginning of a study. In case of missing gender information in ongoing studies, Gender Scores can be used to retrospectively encode gender information.
3. Consider female sex-specific factors in data collection and analysis (e.g., menstrual and reproductive history, contraception, duration of use and types), menopause and menopausal hormone therapy (duration use and types) as well as gender-affirming hormone therapy and gonadectomy for all sexes and genders.
4. The effect of both sex and gender on kidney and CV outcomes should be analyzed, such as:
  - CKD prevalence (eGFR and albuminuria) and progression;
  - initiation and type of dialysis or conservative care;
  - referral to nephrology and CKD management in ambulatory care;
  - referral to transplant waitlist and receipt of a kidney transplant;
  - overall and cardiovascular mortality;
  - blood pressure targets;
  - anemia targets;
  - CKD-MBD targets, fracture risk;
  - medication effects and adverse effects.
5. Finally, sex- and gender-specific information should systematically be incorporated into all Clinical Practice Guidelines.

## Figures and Tables

**Table 1:** Odds Ratios (and 95% confidence intervals) for various chronic diseases in a large LGBTQI+ cohort (n = 30,763) adapted from [59]. Underlying models were adjusted for age (continuous), annual income, employment type, and year of study enrollment.

	Cisgender Sexual Minority Women (Compared to Cisgender Heterosexual Women)	Cisgender Sexual Minority Men (Compared to Cisgender Heterosexual Men)	Gender-Diverse People AFAB ^a^ (Compared to Cisgender Heterosexual Women)	Gender-Diverse People AMAB ^b^ (Compared to Cisgender Heterosexual Men)	Transgender Women (Compared to Cisgender Heterosexual Women)	Transgender Men (Compared to Cisgender Heterosexual Men)
**Number of individuals** (total LGBTQI+ population n = 30,763)	n = 16,096 (52.3%)	n = 10,980 (35.7%)	n = 1433 (4.7%)	n = 482 (1.6%)	n = 849 (2.8%)	n = 923 (3.0%)
**Chronic kidney disease**	**0.83** **(0.73, 0.93)**	**1.06** **(0.97, 1.16)**	**0.92** **(0.58, 1.48)**	**0.37** **(0.15, 0.89)**	**1.11** **(0.79, 1.57)**	**0.87** **(0.61, 1.25)**
Cardiovascular disease	0.98 (0.90, 1.07)	0.99 (0.93, 1.07)	0.99 (0.67, 1.45)	0.83 (0.52, 1.32)	1.10 (0.83, 1.46)	0.68 (0.49, 0.94)
Diabetes	0.85 (0.79, 0.90)	0.91 (0.85, 0.98)	0.62 (0.46, 0.82)	0.69 (0.45, 1.05)	0.87 (0.69, 1.09)	1.17 (0.94, 1.46)
Hypertension	0.91 (0.87, 0.95)	0.99 (0.95, 1.04)	0.78 (0.64, 0.94)	0.69 (0.52, 0.92)	0.95 (0.80, 1.11)	1.06 (0.89, 1.25)
Tobacco use disorder	1.43 (1.32, 1.55)	1.27 (1.15, 1.39)	0.83 (0.56, 1.21)	0.79 (0.45, 1.39)	1.27 (0.92, 1.75)	0.97 (0.69, 1.37)
Anxiety	1.35 (1.29, 1.41)	1.71 (1.61, 1.81)	1.75 (1.51, 2.05)	2.03 (1.57, 2.62)	0.90 (0.74, 1.10)	1.96 (1.62, 2.35)
Depression	1.46 (1.40, 1.53)	1.95 (1.85, 2.07)	2.01 (1.72, 2.34)	2.06 (1.58, 2.68)	1.03 (0.85, 1.25)	2.16 (1.80, 2.59)

^a^ gender-diverse people assigned female at birth, AFAB; ^b^ gender-diverse people assigned male at birth, AMAB.

## Data Availability

No new data were created or analyzed in this study. Data sharing is not applicable to this article.

## Abbreviations

The following abbreviations are used in this manuscript:

ACE	angiotensin-converting enzyme
BIS	Berlin Initiative Study
CKD	Chronic Kidney Disease
CKD-EPI	Chronic Kidney Disease–Epidemiology Collaboration
CVRF	Cardiovascular risk factors
CVD	Cardiovascular disease
DKD	Diabetic kidney disease
EKFC	European Kidney Function Consortium
FAS	Full age spectrum
eGFR	estimated glomerular filtration rate
mGFR	measured glomerular filtration rate
KDIGO	Kidney disease: improving global outcome
LBW	Low birth weight
LGBTQI+	Lesbian, gay, bisexual, transgender, queer, inter individuals
MDRD	Modification in renal diet (study)
NIH	National Institute of Health
OR	Odds Ratio
RCT	Randomized Controlled Trial
SAGER	Sex and gender equity in research
SGLT2	Sodium glucose linked transporter 2
